# Effect of an interprofessional simulation program on patient safety competencies of healthcare professionals in Switzerland: a before and after study

**DOI:** 10.3352/jeehp.2023.20.25

**Published:** 2023-08-28

**Authors:** Sylvain Boloré, Thomas Fassier, Nicolas Guirimand

**Affiliations:** 1 Geneva School of Health Sciences, HES-SO University of Applied Sciences and Arts Western Switzerland, Geneva, Switzerland; 2 CIRNEF Interdisciplinary Education and Training Research Centre, University of Rouen Normandy, Rouen, France; 3 Centre for Interprofessional Simulation, Geneva, Switzerland; 4 Division of Internal Medicine for the Aged, Geneva University Hospitals, Geneva, Switzerland; 5 Faculty of Medicine, University of Geneva, Geneva, Switzerland; Hallym University, Korea

**Keywords:** Continuing education, Interprofessional education, Patient safety, Patient simulation, Program evaluation

## Abstract

**Purpose:**

This study aimed to identify the effects of a 12-week interprofessional simulation program, operated between February 2020 and January 2021, on the patient safety competencies of healthcare professionals in Switzerland.

**Methods:**

The simulation training was based on 2 scenarios of hospitalized patients with septic shock and respiratory failure, and trainees were expected to demonstrate patient safety competencies. A single-group before and after study was conducted after the intervention—simulation program, using a measurement tool (the Health Professional Education in Patient Safety Survey) to measure the perceived competencies of physicians, nurses, and nursing assistants. Out of 57 participants, 37 answered the questionnaire surveys 4 times: 48 hours before the training, followed by post-surveys at 24 hours, 6 weeks, and 12 weeks after the training. The linear mixed effect model was applied for the analysis.

**Results:**

Four components out of 6 perceived patient safety competencies improved at 6 weeks but returned to a similar level before training at 12 weeks. Competencies of “communicating effectively,” “managing safety risks,” “understanding human and environmental factors that influence patient safety,” and “recognize and respond to remove immediate risks of harm” are statistically significant both overall and in the comparison between before the training and 6 weeks after the training.

**Conclusion:**

Interprofessional simulation programs contributed to developing some areas of patient safety competencies of healthcare professionals, but only for a limited time. Interprofessional simulation programs should be repeated and combined with other forms of support, including case discussions and debriefings, to ensure lasting effects.

## Graphical abstract

[Fig f3-jeehp-20-25]

## Introduction

### Background/rationale

At least 1 in 30 hospitalized patients present clinical deterioration criteria that compromise their safety [1]. This type of situation requires teamwork, a high degree of adaptability, and complex decision-making skills to ensure patient safety [2]. Developing healthcare professionals’ competencies in patient safety is of critical importance. The Canadian Patient Safety Institute has developed a model to assist in designing educational programs and evaluating their effectiveness. This model consists of 6 interprofessional competency domains—namely, patient safety culture, teamwork, communication, safety, risk and quality improvement, optimization of human and environmental factors, and recognition, response, and disclosure of adverse events [3]. It is essential to consider these 6 competency domains when assessing the effect of healthcare professionals’ training in patient safety, which is made possible by the Health Professional Education in Patient Safety Survey [4]. Among training strategies to develop patient safety competencies, interprofessional simulation has become increasingly important in recent years [5]. To our knowledge, the Health Professional Education in Patient Safety Survey has been used in 2 studies and has shown overall improvement in all 6 competency domains [6,7]. Brown et al. [6] followed 32 nursing students over 3 consecutive semesters with 128 hours of simulation. They found an overall improvement in all 6 competency domains over time, yet with declines between each set of measurements. Kim and Nam [7] studied 114 nursing students involved in a series of 15 two-hour simulation sessions and found an overall increase of 11% after 8 weeks. Although these studies focused on teamwork, all have only surveyed nursing students in initial or postgraduate education. To our knowledge, no published research has used the Health Professional Education in Patient Safety Survey to study the effect of interprofessional simulation in continuing training.

Long-term retention of competencies after simulation training is a challenge [8]. Moreover, it is important to analyze both the collective effect through cohort means and individual changes before drawing conclusions. Unfortunately, many studies on patient safety competencies are limited to comparing pre- and post-training measures, without considering long-term retention and individual variation [6,9].

### Objectives

The aim of this study was to assess the effect of an interprofessional simulation training on the patient safety competencies of healthcare professionals over time within the context of managing a deteriorating patient. Specifically, the study aimed to address the following research question: How do participants’ scores on the Health Professional Education in Patient Safety Survey evolve at 3 follow-up points after training?

## Methods

### Ethics statement

This study’s protocol was approved by the Regional Research Ethics Committee (CCER-2019-01034). Data collection was carried out after obtaining agreement from the hospital management and department heads. Written informed consent was obtained from all participants.

### Study design

This was a before and after study. It was described according to the TREND statement (https://www.cdc.gov/trendstatement/).

### Setting

The study took place at the Centre for Interprofessional Simulation in Geneva, Switzerland. It was embedded within an interprofessional simulation training program of the Geneva University Hospitals, delivered since 2017 for healthcare professionals working in 2 internal medicine divisions. Due to the coronavirus disease 2019 (COVID-19) pandemic, only 8 of the originally planned 20 sessions were held between February 2020 and January 2021. The questionnaire surveys were administered 4 times: before the interprofessional program, and 3 times afterwards.

### Participants

**Fig. 1.**
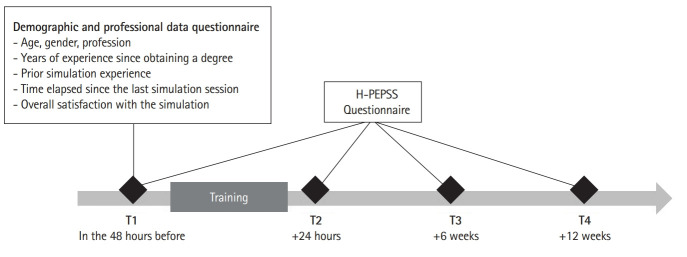
Flow chart of data collection steps for the effect of the interprofessional simulation program on patient safety competencies of healthcare professionals in Switzerland. H-PEPSS, Health Professional Education in Patient Safety Survey.

Each session involved a group of 8 participants: 2 nursing assistants, 4 nurses, and 2 physicians. Participants were enrolled in the interprofessional simulation program as part of their mandatory continuing training. Out of 57 participants, 37 answered the questionnaires 4 times. Participants independently completed surveys online using LimeSurvey ver. 3.20 (LimeSurvey GmbH; https://www.limesurvey.org/). To ensure anonymity, participants generated a personal identification code. No incentives were offered for participation in the study. The survey was sent to participants at 4 points in time: (T1) 48 hours before the training, followed by post-surveys at 24 hours (T2), 6 weeks (T3), and 12 weeks (T4) after the training (Fig. 1).

### Intervention

The training was based on 2 scenarios of hospitalized patients with rapid clinical deterioration (1 scenario involving septic shock and 1 involving respiratory failure), and trainees were expected to demonstrate patient safety competencies. The interprofessional simulation training lasted a total of 4 hours and included briefing, simulation, debriefing (3-phase structure: reaction-analysis-summary), and take-home message activities. Participants played their respective roles.

### Outcomes

The outcome variables were demographic characteristics of participants and 31 questionnaire items of the Health Professional Education in Patient Safety Survey [10].

### Data sources/measurement

The Health Professional Education in Patient Safety Survey is a reliable tool with high internal consistency as suggested by a Cronbach α greater than 0.8 for each of the 6 sections in the original description [4] and a McDonald ω coefficient 0.89 in its French-language version [10]. The French-language version was used in the present study (Supplement 1). The survey consisted of 2 sets of questions. The first set of questions was designed to characterize the sample. The second set of questions used the French-language version of the Health Professional Education in Patient Safety Survey [10]. The demographic and professional data collected included age, gender, profession, years of experience since obtaining a degree, prior simulation experience, time elapsed since the last simulation session, and overall satisfaction with the simulation. The Health Professional Education in Patient Safety Survey questionnaire consists of 5-point Likert scale 38 items divided into 4 sections [4]. The first section contains a 4-item subscale related to clinical safety. The second section contains 23 items for the 6 domains based on the Canadian Patient Safety Competencies Framework [3] through 6 competency subscales: (C1) working in teams with other health professionals (6 items), (C2) communicating effectively (3 items), (C3) managing safety risks (3 items), (C4) understanding human and environmental factors (3 items), (C5) identifying and responding to eliminate immediate risks of harm (4 items), and (C6) culture of safety (4 items). The third section contains 7 items related to safety in health professional education. The final section contains a 4-item subscale related to feeling competent to discuss patient safety. Only sections 1, 2, and 4 were used in this study, as section 3 is intended for students and not professionals. A score between 0 and 40 points was obtained, representing the combined average score of the 8 selected subscales. Raw response data from participants are available in Dataset 1. Coded data are available in Dataset 2.

### Bias

Convenience sampling can be biased because it means that certain people are less likely to be included than others.

### Study size

Sample size estimation was not done. All target population is included.

### Assignment method

Only 1 group was followed up.

### Blinding (masking)

No blinding was done.

### Unit of analysis

The unit of analysis was the single group.

### Statistical methods

Data were analyzed using Excel ver. 16.52 (Microsoft Corp.) and STATA ver. 15.1 (Stata Corp.). Descriptive statistics summarized the distribution, central tendency, and dispersion of responses. For continuous variables, the distribution of data was first evaluated for normality using the Shapiro-Wilk test. However, all the variables did not pass the Shapiro-Wilk test, we additionally checked a q-q plot, which did not show marked deviation from linearity. We therefore assumed that normal distribution assumption for parametric test was not violated, and decided to apply linear mixed effect model, which was made with times (T1, T2, T3, and T4) as independent fixed factors, and individual patients as random effects. Significance was set at α=0.05.

## Results

### Participants/recruitment/baseline data/numbers analyzed

Out of the 64 planned participants, 57 were able to attend the training and 37 (64.91%) responded to the survey at least once (Table 1). The nursing assistant group consisted exclusively of women, whereas the nursing group had more women, and the physician group was majority men. Physicians generally had fewer years of experience. Almost 3-quarters of the learners had previously participated in simulation training, and they reported high satisfaction.

### Baseline equivalence

Equivalence testing could not be done since there was no control group.

### Outcomes and estimation

**Fig. 2.**
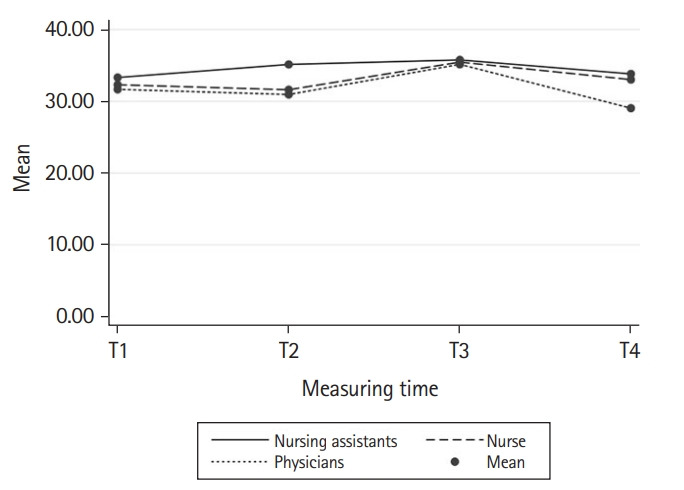
Changes in the combined score by profession for the effect of the interprofessional simulation program on patient safety competencies of healthcare professionals in Switzerland.

Between T1 and T2, the combined score of the measurement tools remained stable (Table 2, Dataset 3). The progression curve of the combined score categorized by profession also showed the highest score at T3 (Fig. 2). While a decrease is observed for physicians at T4, the curve flattened for nurses and nursing assistants.

The results of linear mixed effect model showed overall statistically significant difference in “communicating effectively (C3),” managing safety risks (C4),” “understanding human and environmental factors that influence patient safety (C5),” and “recognize and respond to remove immediate risks of harm (C6)” (P=0.028, P=0.002, P=0.002, and P=0.009, respectively). The scores of C3, C4, C5, and C6 at 6 weeks after intervention (T3) are significantly higher than those at just before interventin (T1) (P=0.008, P=0.001, P=0.005, and P=0.011, respectively). There was no significant change over time for the score of the sections, including “practice of clinical safety,” “culture of safety,” “working in teams with other health professionals,” and “speaking up about patient safety (Table 3).”

### Ancillary analyses

There was no other analysis.

### Adverse events

There were no adverse events.

## Discussion

### Key results

The aim of this study was to assess the effect of interprofessional simulation training on patient safety competencies of individual trainees through the management of a deteriorating patient. A significant temporary increase at 6 weeks after the intervention in perceived competencies related to 4 components of patient safety was observed among physicians, nurses, and nursing assistants, suggesting a return to pre-training levels. These changes should be seen as trends.

### Interpretation

Four out of all 6 patient safety competencies from the framework developed by Canadian Patient Safety Institute [3] showed a peak of positive growth at T3, and higher than those at T1. Competencies 3, 4, 5, and 6 had a significant difference between T1 and T3. Competency 4, “managing safety risks,” which involves “recognizing routine situations in which safety problems may arise,” “identifying and implementing safety solutions,” and “anticipating and managing high risk situations,” is indeed consistent with managing simulated clinical deterioration scenarios [2]. Competency 5, “understanding human and environmental factors,” makes sense in light of the discussions held during debriefings about collaboration and the human resources to rely on.

In addition to these 6 competencies, the Health Professional Education in Patient Safety Survey questionnaire focuses on speaking out on patient safety. This section had a no difference after training than before training. While this topic should be addressed during such interprofessional simulation activities, it may not receive sufficient emphasis in the studied program. The need for debate and deeper understanding of these issues suggests considering other approaches where supervisory staff could be involved, given the responsibility for addressing errors made by oneself or identified by others.

### Comparison with previous studies

Comparing the results of our research with those of other studies is complicated by the specificity of the measurement instruments used. Although they fall within the domain of self-efficacy, the precise moments of measurement and their multiplicity make comparisons difficult. Our results for self-efficacy before and after simulation-based training are consistent with other studies that showed a significant improvement [11-13]. However, when it comes to longitudinal follow-up, studies on self-efficacy in cardiopulmonary resuscitation procedures have shown an increase in self-efficacy 6 months after training [14], unlike our results at 3 months; however, those studies considered different indicators. Two studies have confirmed a positive improvement in competencies, as assessed by the Health Professional Education in Patient Safety Survey questionnaire, following simulation-based training. The study by Kim and Nam [7] was limited to a pre-post measurement at 8 weeks. Results similar to ours between T1 and T3 were observed. In the follow-up measurements reported by Brown et al. [6], with multiple simulation trainings, an overall improvement was seen, but there were declines between each set of measurements. These studies only included nursing students, whereas our research offers new information in the context of continuing training through interprofessional simulation.

### Limitations/generalizability

Due to the interruptions caused by the COVID-19 pandemic and the subsequent cancellation of training sessions, data collection was initiated but faced challenges, leading to a high rate of missing data for follow-up questionnaires. Although the study design was a single-group before and after study, the nonparametric Mann-Whitney U test for independent samples or repeated measures analysis of variance could not be used because there were too much missing data. There are methods available to impute missing data, but due to the high amount of missing data, it is not advisable to do so. The linear mixed-effects model is the only way to account for missing data The small number of participants from each profession and the convenience sample of this pilot study limit its generalizability. In particular, the comparison of influencing factors between professions was not done due to small number of each group. Furthermore, self-assessment tools have limitations because they focus on measuring an individual’s perception of their abilities. They can help trainers identify learners who may have difficulties in practical situations, but they cannot prevent biases, such as the Dunning-Kruger effect—namely, the tendency of people with low ability in a specific area to give overly positive assessments of this ability—and they cannot accurately determine the correspondence between a learner’s perception and their actual professional competencies.

### Suggestions

This study has several implications. This study showed the importance of selecting interprofessional simulation scenarios that promote the development of patient safety competencies, specifically by utilizing scenarios involving the management of deteriorated patients. The utility of longitudinal design to examine the effect of simulation training is critical, ideally with randomization across multiple centers. The choice of measurement placements appeared relevant considering the observed trends [8]. Given the no significant difference in results between the T1 and T2 responses, the number of measurements in such a follow-up could be limited to those taken at 6, 12, or even 18 weeks. Finally, the way in which groups of professionals are assembled for training sessions needs to be considered. In continuing training, opportunities to learn within an interprofessional team are rare. For learning to be optimized, it is beneficial for participants to come from the same care unit, rather than from several units within a common department.

### Conclusion

The results of this research confirm that an interprofessional simulation for managing situations of clinical deterioration contributed to the development of some patient safety competency domains, although the effects were limited in time. “Comfort speaking up about patient safety” should be better integrated given its importance. To ensure lasting effects, interprofessional simulations should be repeated and integrated with other forms of support, including case discussions and debriefings. These activities encourage reflection on patient safety incidents and aid in the development of preventive strategies. As self-efficacy measures are inherently subjective, it is important to examine individual changes in detail, in addition to overall results, when using such measures.

## Figures and Tables

**Figure f3-jeehp-20-25:**
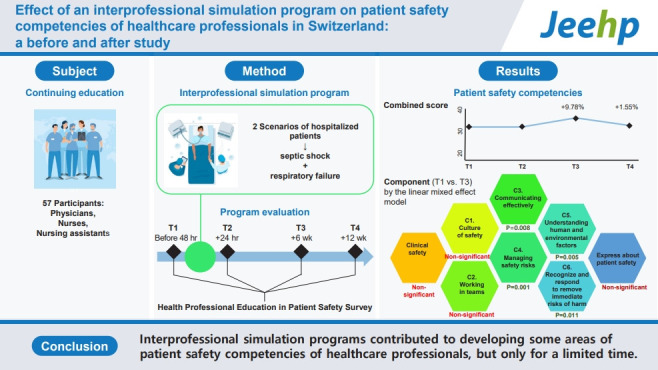


**Table 1.** Demographic and professional data of the respondents (N=37)

Characteristic	Nursing assistants (n=9)			Nurses (n=18)			Physicians (n=10)		
	%	Mean±SD	Min–max	%	Mean±SD	Min–max	%	Mean±SD	Min–max
Age (yr)		35.60±10.16	25–51		37.17±11.37	24–54		34.50±8.83	28–49
Gender, female	100			88.89			40		
Years of experience (yr)		13.38±12.02	2.83–31.42		11.48±12.63	0.17–31.83		7.10±8.07	1.25–22.00
Experience of simulation	80			75			66.67		
Time since last simulation date (yr)		1.04±0.75	0.17–2.00		1.37±0.79	0.50–3.00		0.83±0.79	0.25–2.00
Satisfaction in simulation^a)^		4.00±0.00	4–4		3.67±0.50	3–4		3.50±0.58	3–4

SD, standard deviation; Min, minimum; Max, maximum.

a) Range from 1 to 5: strongly disagree/somewhat disagree/shared/somewhat agree/agree.

**Table 2.** Combined score results for all respondents

Time	No.	Mean	Min–max
T1	19	32.35	26.92–38.33
T2	24	32.3	27.5–39.5
T3	15	35.46	30.33–40
T4	18	32.85	26.75–38.83

Min, minimum; Max, maximum; T1, just before; T2, just after; T3, 6 weeks; T4, 12 weeks.

**Table 3.** Results of linear mixed effect model for each component for all respondents at the 4 measurement times

Component	Overall	T1 vs. T2	T1 vs. T3	T1 vs. T4
	P-value	P-value	P-value	P-value
Practice of clinical safety	0.402			
C1. Culture of safety	0.196			
C2. Working in teams with other health professionals	0.100			
C3. Communicating effectively	**0.028**	0.596	**0.008**	0.081
C4. Managing safety risks	**0.002**	0.871	**0.001**	0.058
C5. Understanding human and environmental factors that influence patient safety	**0.002**	0.635	**0.005**	0.267
C6. Recognize and respond to remove immediate risks of harm	**0.009**	0.561	**0.011**	0.236
Speaking up about patient safety	0.706			

Statistically significant results are marked in bold.

## Footnotes

**Data availability**
Data files are available from Harvard Dataverse: https://doi.org/10.7910/DVN/BJUHAG Dataset 1. Raw response data from participants.

Dataset 2. Coded data for analysis.

Dataset 3. Results of statistical analyses.

## Supplementary materials

Supplementary files are available from Harvard Dataverse: https://doi.org/10.7910/DVN/BJUHAG

Supplement 1. French-language version of the Health Professional Education in Patient Safety Survey.

Supplement 2. Audio recording of the abstract.
