# Supplementary material for: Effect of an interprofessional simulation program on patient safety competencies of healthcare professionals in Switzerland: a before and after study
Source: J Educ Eval Health Prof. 2023 Aug 28;20:25. doi: 10.3352/jeehp.2023.20.25 (PMC10519772; doi:10.3352/jeehp.2023.20.25)
Supplement: Supplementary file 4 — Supplement 1. French-language version of the Health Professional Education in Patient Safety Survey. [file jeehp-20-25-suppl.docx]

**Supplement 1**

French-language version of the H-PEPSS

**Section 1 : Sécurité dans la pratique clinique**

**La sécurité dans la clinique: « Je me sens compétent pour …**

**1. Appliquer les règles d’hygiène des mains**

Pas du tout d’accord ❒ Plutôt pas d’accord ❒ Je suis partagé(e) ❒ Plutôt d’accord ❒ Tout à fait d’accord ❒ Je ne sais pas❒

**2. Gérer la prévention des infections**

Pas du tout d’accord ❒ Plutôt pas d’accord ❒ Je suis partagé(e) ❒ Plutôt d’accord ❒ Tout à fait d’accord ❒ Je ne sais pas❒

**3. Gérer la médication de manière sécuritaire**

Pas du tout d’accord ❒ Plutôt pas d’accord ❒ Je suis partagé(e) ❒ Plutôt d’accord ❒ Tout à fait d’accord ❒ Je ne sais pas❒

**4. Réaliser des pratiques cliniques en toute sécurité, en général**

Pas du tout d’accord ❒ Plutôt pas d’accord ❒ Je suis partagé(e) ❒ Plutôt d’accord ❒ Tout à fait d’accord ❒ Je ne sais pas❒

**Section 2 : Compétences en sécurité des patients**

**C1. Le travail d’équipe avec d’autres professionnels de la santé: « Je me sens compétent pour …**

**5. Gérer des dynamiques de groupe en tenant compte des différences hiérarchiques**

Pas du tout d’accord ❒ Plutôt pas d’accord ❒ Je suis partagé(e) ❒ Plutôt d’accord ❒ Tout à fait d’accord ❒ Je ne sais pas❒

**6. Gérer des conflits interprofessionnels**

Pas du tout d’accord ❒ Plutôt pas d’accord ❒ Je suis partagé(e) ❒ Plutôt d’accord ❒ Tout à fait d’accord ❒ Je ne sais pas❒

**7. Soutenir les membres de l’équipe lors d’un événement indésirable ou d’un accident évité de justesse**

Pas du tout d’accord ❒ Plutôt pas d’accord ❒ Je suis partagé(e) ❒ Plutôt d’accord ❒ Tout à fait d’accord ❒ Je ne sais pas❒

**8. Impliquer le patient dans ses soins**

Pas du tout d’accord ❒ Plutôt pas d’accord ❒ Je suis partagé(e) ❒ Plutôt d’accord ❒ Tout à fait d’accord ❒ Je ne sais pas❒

**9. Partager la responsabilité, le leadership et la prise de décisions**

Pas du tout d’accord ❒ Plutôt pas d’accord ❒ Je suis partagé(e) ❒ Plutôt d’accord ❒ Tout à fait d’accord ❒ Je ne sais pas❒

**10. Encourager les membres de l’équipe à oser s’exprimer, à poser des questions, à relever des défis, à formuler des recommandations et à prendre leurs responsabilités sur les questions de sécurité**

Pas du tout d’accord ❒ Plutôt pas d’accord ❒ Je suis partagé(e) ❒ Plutôt d’accord ❒ Tout à fait d’accord ❒ Je ne sais pas❒

**C2. La communication efficace: « Je me sens compétent pour …**

**11. Communiquer de manière cohérente et compréhensible auprès des patients afin d’améliorer leur sécurité**

Pas du tout d’accord ❒ Plutôt pas d’accord ❒ Je suis partagé(e) ❒ Plutôt d’accord ❒ Tout à fait d’accord ❒ Je ne sais pas❒

**12. Communiquer de manière efficace avec l’équipe soignante afin d’améliorer la sécurité des patients**

Pas du tout d’accord ❒ Plutôt pas d’accord ❒ Je suis partagé(e) ❒ Plutôt d’accord ❒ Tout à fait d’accord ❒ Je ne sais pas❒

**13. Communiquer, de manière verbale et non verbale, de façon efficace afin d’éviter les événements indésirables**

Pas du tout d’accord ❒ Plutôt pas d’accord ❒ Je suis partagé(e) ❒ Plutôt d’accord ❒ Tout à fait d’accord ❒ Je ne sais pas❒

**C3. La gestion des risques de sécurité: « Je me sens compétent pour …**

**14. Reconnaître des situations routinières où des problèmes de sécurité pourraient survenir**

Pas du tout d’accord ❒ Plutôt pas d’accord ❒ Je suis partagé(e) ❒ Plutôt d’accord ❒ Tout à fait d’accord ❒ Je ne sais pas❒

**15.Identifier et mettre en pratique des solutions sécuritaires**

Pas du tout d’accord ❒ Plutôt pas d’accord ❒ Je suis partagé(e) ❒ Plutôt d’accord ❒ Tout à fait d’accord ❒ Je ne sais pas❒

**16. Anticiper et gérer des situations à risque élevé**

Pas du tout d’accord ❒ Plutôt pas d’accord ❒ Je suis partagé(e) ❒ Plutôt d’accord ❒ Tout à fait d’accord ❒ Je ne sais pas❒

**C4. La compréhension des facteurs humains et environnementaux: « Je me sens compétent pour …**

**17. Anticiper les effets de facteurs humains, tel que la fatigue, qui pourraient nuire à la sécurité du patient**

Pas du tout d’accord ❒ Plutôt pas d’accord ❒ Je suis partagé(e) ❒ Plutôt d’accord ❒ Tout à fait d’accord ❒ Je ne sais pas❒

**18. Utiliser de manière sécuritaire la technologie dans le domaine de la santé**

Pas du tout d’accord ❒ Plutôt pas d’accord ❒ Je suis partagé(e) ❒ Plutôt d’accord ❒ Tout à fait d’accord ❒ Je ne sais pas❒

**19. Anticiper les effets des facteurs environnementaux** **tels que la charge de travail, l’ergonomie, les ressources, qui pourraient mettre à risque la sécurité du patient**

Pas du tout d’accord ❒ Plutôt pas d’accord ❒ Je suis partagé(e) ❒ Plutôt d’accord ❒ Tout à fait d’accord ❒ Je ne sais pas❒

**C5. Reconnaître, réagir et divulguer des événements indésirables: « Je me sens compétent pour …**

**20. Reconnaître un événement indésirable ou un accident évité de justesse**

Pas du tout d’accord ❒ Plutôt pas d’accord ❒ Je suis partagé(e) ❒ Plutôt d’accord ❒ Tout à fait d’accord ❒ Je ne sais pas❒

**21. Éviter de nuire au patient et aux autres personnes impliquées dans l’événement en agissant immédiatement quand les risques se présentent**

Pas du tout d’accord ❒ Plutôt pas d’accord ❒ Je suis partagé(e) ❒ Plutôt d’accord ❒ Tout à fait d’accord ❒ Je ne sais pas❒

**22. Annoncer la survenue d’un événement indésirable au patient**

Pas du tout d’accord ❒ Plutôt pas d’accord ❒ Je suis partagé(e) ❒ Plutôt d’accord ❒ Tout à fait d’accord ❒ Je ne sais pas❒

**23. Participer, en temps opportun, à l’analyse d’un événement indésirable, ainsi qu’à la réflexion et la planification d’actions à mener pour éviter qu’il ne se reproduise**

Pas du tout d’accord ❒ Plutôt pas d’accord ❒ Je suis partagé(e) ❒ Plutôt d’accord ❒ Tout à fait d’accord ❒ Je ne sais pas❒

**C6. La culture de sécurité : « Je me sens compétent pour …**

**24. Appréhender la complexité des soins et tenir compte de ce qui la rend difficile (l’aménagement du lieu de travail, les ressources humaines disponibles, la technologie, les limites humaines)**

Pas du tout d’accord ❒ Plutôt pas d’accord ❒ Je suis partagé(e) ❒ Plutôt d’accord ❒ Tout à fait d’accord ❒ Je ne sais pas❒

**25. Poser des questions et oser s’exprimer lorsqu’on observe des pratiques risquées**

Pas du tout d’accord ❒ Plutôt pas d’accord ❒ Je suis partagé(e) ❒ Plutôt d’accord ❒ Tout à fait d’accord ❒ Je ne sais pas❒

**26. Encourager les patients et l’équipe soignante à exprimer leurs inquiétudes par rapport aux pratiques risquées**

Pas du tout d’accord ❒ Plutôt pas d’accord ❒ Je suis partagé(e) ❒ Plutôt d’accord ❒ Tout à fait d’accord ❒ Je ne sais pas❒

**27. Anticiper les effets des systèmes de santé (par exemple : l’organisation et la gestion du travail, le milieu de travail, les politiques, les ressources, la communication et autres procédures) en cas d’événements indésirables**

Pas du tout d’accord ❒ Plutôt pas d’accord ❒ Je suis partagé(e) ❒ Plutôt d’accord ❒ Tout à fait d’accord ❒ Je ne sais pas❒

**Section 3 : La sécurité des patients dans la formation des professionnels de santé**

**En répondant aux questions suivantes, veuillez svp considérer votre formation globale en tant que professionnel de santé (les cours théoriques, les stages, et les ateliers de simulation).**

**28. En tant qu’étudiant(e), l’étendue de ma propre pratique professionnelle est très claire pour moi**

Pas du tout d’accord ❒ Plutôt pas d’accord ❒ Je suis partagé(e) ❒ Plutôt d’accord ❒ Tout à fait d’accord ❒

**29. Les questions entourant la sécurité des patients sont traitées de la même façon par les différents enseignants**

Pas du tout d’accord ❒ Plutôt pas d’accord ❒ Je suis partagé(e) ❒ Plutôt d’accord ❒ Tout à fait d’accord ❒

**30. Les occasions d’apprendre et d’interagir avec les membres de l’équipe pluridisciplinaire sont suffisamment fréquentes**

Pas du tout d’accord ❒ Plutôt pas d’accord ❒ Je suis partagé(e) ❒ Plutôt d’accord ❒ Tout à fait d’accord ❒

**31. Je comprends bien que parler des événements indésirables peut modifier la pratique quotidienne du soin et réduire la fréquence de leur survenue**

Pas du tout d’accord ❒ Plutôt pas d’accord ❒ Je suis partagé(e) ❒ Plutôt d’accord ❒ Tout à fait d’accord ❒

**32. Le sujet de la sécurité des patients est bien intégré dans l’ensemble du programme de formation**

Pas du tout d’accord ❒ Plutôt pas d’accord ❒ Je suis partagé(e) ❒ Plutôt d’accord ❒ Tout à fait d’accord ❒

**33. Les éléments cliniques de la sécurité des patients (par exemple : l’hygiène des mains, le transfert des patients, l’administration sécuritaire des médicaments) sont bien abordés dans notre programme**

Pas du tout d’accord ❒ Plutôt pas d’accord ❒ Je suis partagé(e) ❒ Plutôt d’accord ❒ Tout à fait d’accord ❒

**34. Les éléments systémiques qui favorisent la sécurité des patients sont bien abordés dans notre programme (par exemple : l’organisation, la gestion, le milieu de travail, les politiques, les ressources, la communication et autres procédures)**

Pas du tout d’accord ❒ Plutôt pas d’accord ❒ Je suis partagé(e) ❒ Plutôt d’accord ❒ Tout à fait d’accord ❒

**Section 4 : S’exprimer au sujet de la sécurité des patients**

**Comment vous sentez-vous actuellement par rapport aux énoncés suivants :**

**35. Quand je vois un membre de l’équipe agir de façon non sécuritaire en milieu clinique je me sens à l’aise de lui en parler**

Pas du tout d’accord ❒ Plutôt pas d’accord ❒ Je suis partagé(e) ❒ Plutôt d’accord ❒ Tout à fait d’accord ❒

**36. Je crains les répercussions si je commets une erreur grave**

Pas du tout d’accord ❒ Plutôt pas d’accord ❒ Je suis partagé(e) ❒ Plutôt d’accord ❒ Tout à fait d’accord ❒

**37. Je trouve difficile de questionner les décisions ou les actions des supérieurs hiérarchiques**

Pas du tout d’accord ❒ Plutôt pas d’accord ❒ Je suis partagé(e) ❒ Plutôt d’accord ❒ Tout à fait d’accord ❒

**38. Dans la pratique clinique, la discussion autour des événements indésirables met plus l’accent sur la responsabilité des éléments systémiques que sur celles des individu(s) impliqués dans l’événement en question**

Pas du tout d’accord ❒ Plutôt pas d’accord ❒ Je suis partagé(e) ❒ Plutôt d’accord ❒ Tout à fait d’accord ❒
